# Acute Insulin Stimulation Induces Phosphorylation of the Na-Cl Cotransporter in Cultured Distal mpkDCT Cells and Mouse Kidney

**DOI:** 10.1371/journal.pone.0024277

**Published:** 2011-08-31

**Authors:** Eisei Sohara, Tatemitsu Rai, Sung-Sen Yang, Akihito Ohta, Shotaro Naito, Motoko Chiga, Naohiro Nomura, Shih-Hua Lin, Alain Vandewalle, Eriko Ohta, Sei Sasaki, Shinichi Uchida

**Affiliations:** 1 Department of Nephrology, Graduate School of Medicine, Tokyo Medical and Dental University, Tokyo, Japan; 2 Division of Nephrology, Department of Medicine, Tri-Service General Hospital, Taipei, Taiwan; 3 INSERM U773, Paris; Université Paris 7 -Deni Diderot, Paris, France; University of Houston, United States of America

## Abstract

The NaCl cotransporter (NCC) is essential for sodium reabsorption at the distal convoluted tubules (DCT), and its phosphorylation increases its transport activity and apical membrane localization. Although insulin has been reported to increase sodium reabsorption in the kidney, the linkage between insulin and NCC phosphorylation has not yet been investigated. This study examined whether insulin regulates NCC phosphorylation. In cultured mpkDCT cells, insulin increased phosphorylation of STE20/SPS1-related proline-alanine-rich kinase (SPAK) and NCC in a dose-dependent manner. This insulin-induced phosphorylation of NCC was suppressed in WNK4 and SPAK knockdown cells. In addition, Ly294002, a PI3K inhibitor, decreased the insulin effect on SPAK and NCC phosphorylation, indicating that insulin induces phosphorylation of SPAK and NCC through PI3K and WNK4 in mpkDCT cells. Moreover, acute insulin administration to mice increased phosphorylation of oxidative stress-responsive kinase-1 (OSR1), SPAK and NCC in the kidney. Time-course experiments in mpkDCT cells and mice suggested that SPAK is upstream of NCC in this insulin-induced NCC phosphorylation mechanism, which was confirmed by the lack of insulin-induced NCC phosphorylation in SPAK knockout mice. Moreover, insulin administration to WNK4 hypomorphic mice did not increase phosphorylation of OSR1, SPAK and NCC in the kidney, suggesting that WNK4 is also involved in the insulin-induced OSR1, SPAK and NCC phosphorylation mechanism *in vivo*. The present results demonstrated that insulin is a potent regulator of NCC phosphorylation in the kidney, and that WNK4 and SPAK are involved in this mechanism of NCC phosphorylation by insulin.

## Introduction

The NaCl cotransporter (NCC) is expressed in the distal convoluted tubules (DCT) and plays a major role in renal electrolyte balance [1]. As previously reported [2], [3], it is well known that NCC phosphorylation results in increased NCC localization at the cell membrane and increased cotransporter activity. Recently, several physiological regulators of NCC phosphorylation have been reported. We have reported that NCC phosphorylation was increased by a low-salt diet and decreased by a high-salt diet, through regulation by aldosterone, which is a strong regulator of NCC phosphorylation [4]. In addition, we and other groups found that angiotensin II is another regulator of NCC phosphorylation [5], [6], [7]. Moreover, Vallon et al. reported that administration of either a low-sodium or a low-potassium diet increased NCC phosphorylation in mice through serum and glucocorticoid inducible kinase 1 (SGK1) [8]. Vasopressin has also been reported as a regulator of NCC phosphorylation [2].

Insulin is known to regulate renal sodium reabsorption. In fact, insulin infusion increases sodium reabsorption in the kidney [9], [10], [11], [12]. Although it has been reported that insulin increases the activity of epithelial Na channel (ENaC) in the collecting duct [11], [12], a linkage between insulin and NCC phosphorylation has not been reported yet.

Pseudohypoaldosteronism type II (PHAII) is an autosomal dominant disease characterized by hypertension due to increased renal salt reabsorption, and hyperkalemia [13], [14], [15]. Mutations in with-no-lysine kinase 1 (WNK1) and with-no-lysine kinase 4 (WNK4) have been shown to cause PHAII [16]. Recently, we generated *WNK4^D561A/+^* knock-in mice, an ideal mouse model of PHAII, and found that the pathogenesis of PHAII is the constitutive activation of the WNK-OSR1/SPAK kinases-NaCl cotransporter (NCC) cascade, resulting in gain of function of NCC [3]. In both OSR1 and SPAK kinase-dead knock-in and SPAK knockout, it was recently demonstrated that OSR1 and SPAK kinases have a major role in NCC phosphorylation in the kidney [17], [18].

In this study, we investigated whether insulin regulates the NCC phosphorylation. Acute insulin stimulation was found to increase SPAK and NCC phosphorylation in cultured mouse distal convoluted mpkDCT cells [7], [19]. Furthermore, increased phosphorylation of OSR1, SPAK and NCC in insulin-administered mice kidney was also confirmed. This insulin-stimulated OSR1, SPAK and NCC phosphorylation was not found in the kidneys from WNK4 hypomorphic and SPAK knockout mice, respectively, indicating that WNK4 and SPAK are involved in NCC phosphorylation by insulin *in vivo*. These results indicate that insulin is a substantial regulator of the WNK-OSR1/SPAK-NCC phosphorylation cascade *in vivo* in the kidney.

## Results

### Insulin increased phosphorylation of SPAK and NCC in mpkDCT cells

**Figure 1:**
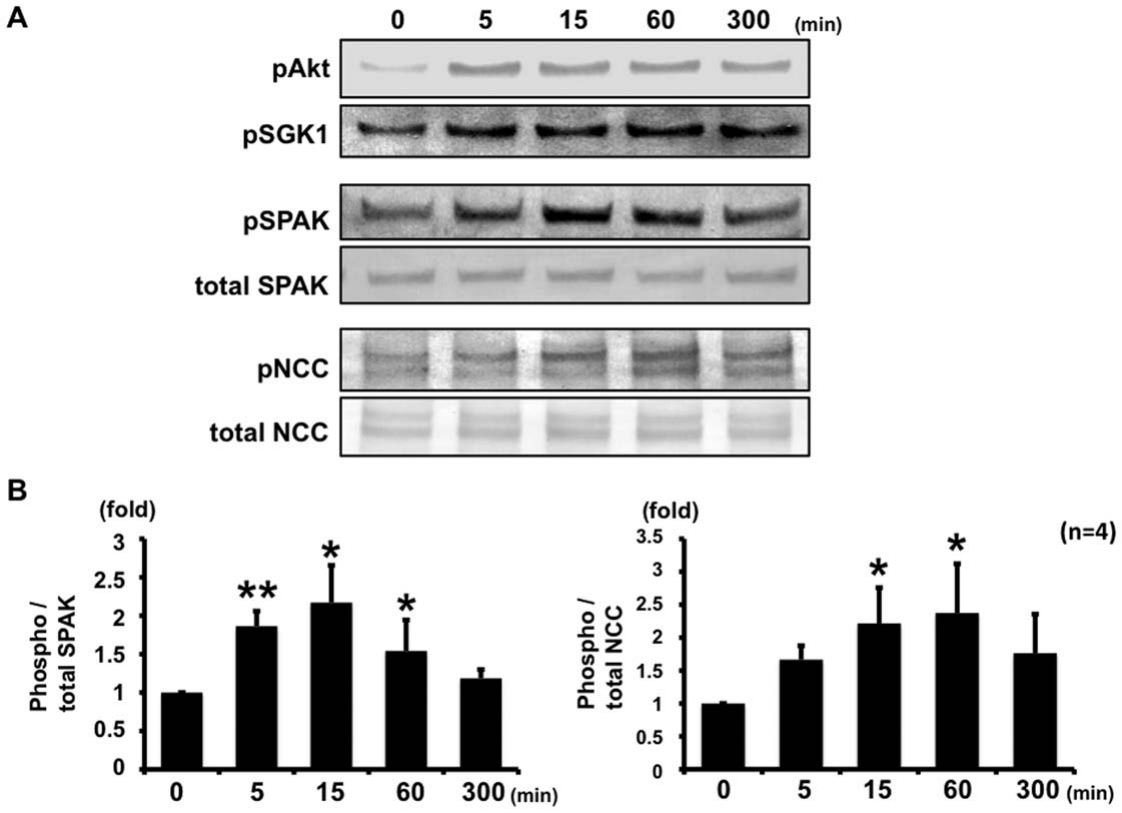
Time-course of insulin-stimulated SPAK and NCC phosphorylation in mpkDCT cells. A. Representative blots of phosphorylation of SPAK and NCC by 100 nM insulin in mpkDCT cells. Insulin increased phosphorylation of SPAK and NCC, as well as Akt and SGK1. B. Densitometry analysis of phosphorylation of NCC by insulin in mpkDCT cells. Values (n = 4) are expressed as the ratio to the signals in insulin-free samples. The level of SPAK phosphorylation increased to the maximal extent at 15 min and returned to the basal level after 300 min. On the other hand, the level of NCC phosphorylation increased to the maximal extent at 60 min. *P<0.05, **P<0.01.

**Figure 2:**
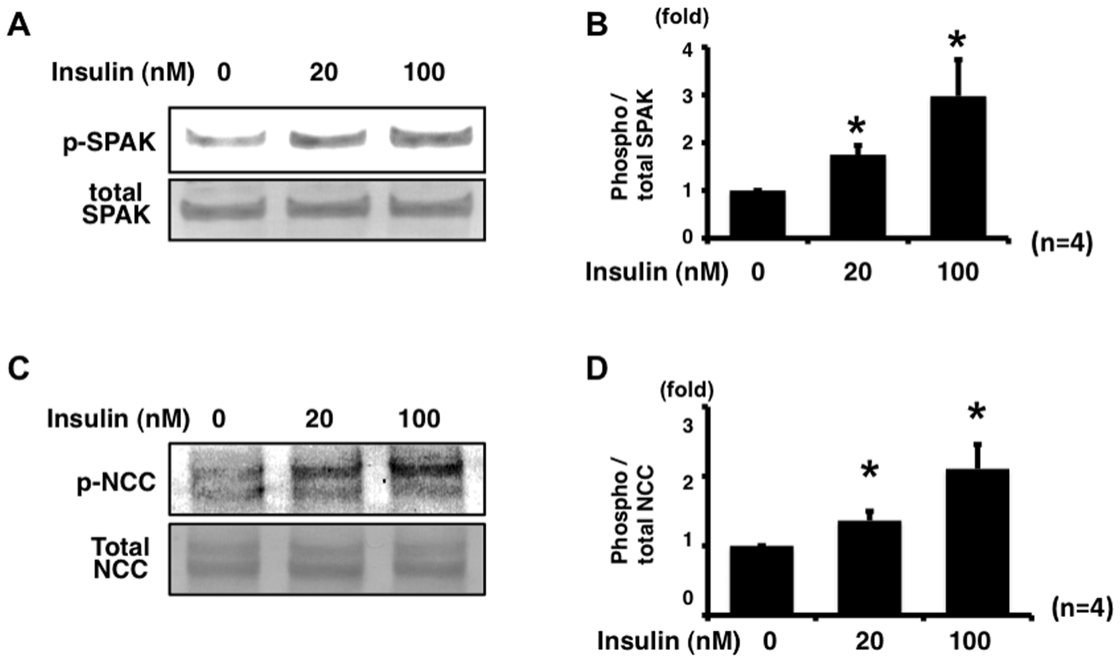
Insulin phosphorylates NCC and SPAK in mpkDCT cells in a dose-dependent manner. A. Representative blot of phosphorylation of SPAK by insulin in mpkDCT cells. mpkDCT cells were incubated with insulin for 60 min at 20 nM and 100 nM. Insulin significantly increased phosphorylation of endogenous SPAK, compared to insulin-free control, in a dose-dependent manner. B. Densitometry analysis of phosphorylation of SPAK by insulin in mpkDCT cells. Values (n = 4) are expressed as the ratio to the signals in insulin-free samples. *P<0.05. C. Representative blot of phosphorylation of NCC by insulin in mpkDCT cells. Insulin significantly increased phosphorylation of endogenous NCC, compared to insulin-free control, in a dose-dependent manner. D. Densitometry analysis of phosphorylation of NCC by insulin in mpkDCT cells. Values (n = 4) are expressed as the ratio to the signals in insulin-free samples. *P<0.05.

To determine whether insulin regulates NCC phosphorylation, mpkDCT cells that endogenously express OSR1, SPAK and NCC were used. First, to evaluate the effect of insulin on SPAK and NCC phosphorylation in mpkDCT cells, a time-course experiment after the stimulation by insulin was performed (Figure 1). Five minutes after insulin addition, there was a significant increase in SPAK phosphorylation levels, which increased to maximum levels 15 min after insulin stimulation and declined to basal levels over the following 6 h. However, expression and phosphorylation of OSR1 were not increased in mpkDCT cells by 100 nM insulin (data not shown). Similar to SPAK, NCC phosphorylation increased after insulin stimulation. However, after insulin stimulation, increased NCC phosphorylation in mpkDCT cells reached the maximum levels at 60 min, indicating that increased NCC phosphorylation occurred after increased SPAK phosphorylation and suggesting that SPAK is upstream of NCC in the phosphorylation cascade in this insulin-induced NCC phosphorylation mechanism. It was also confirmed that insulin-stimulated phosphorylation of SPAK and NCC was dose-dependent in mpkDCT cells (Figure 2).

**Figure 3:**
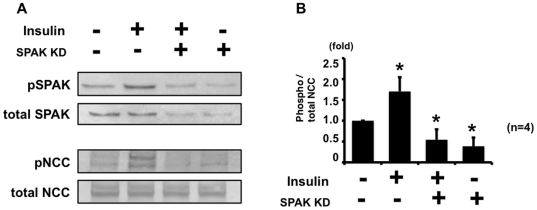
Insulin-induced phosphorylation of NCC was impaired in SPAK knockdown mpkDCT cells. A. Representative blot of phosphorylation of NCC by insulin in SPAK knockdown mpkDCT cells. mpkDCT cells were incubated with insulin for 60 min at 100 nM. Insulin-induced NCC phosphorylation was impaired in SPAK knockdown mpkDCT cells, although insulin increased phosphorylation of NCC in negative control siRNA-transfected cells. B. Densitometry analysis of phosphorylation of NCC by insulin in negative control and SPAK knockdown mpkDCT cells. Values (n = 4) are expressed as the ratio to the signals in insulin-free negative control siRNA-transfected cells.

**Figure 4:**
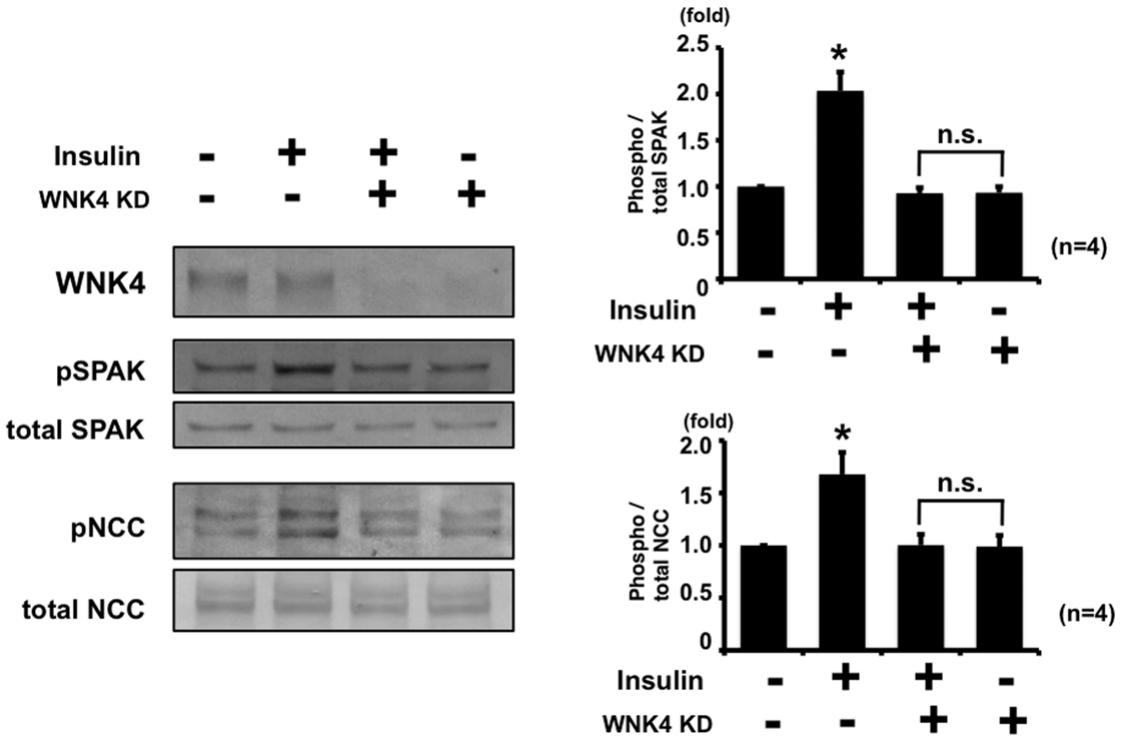
Insulin-induced phosphorylation of NCC was impaired in WNK4 knockdown mpkDCT cells. A. Representative blot of phosphorylation of NCC by insulin in WNK4 knockdown mpkDCT cells. mpkDCT cells were incubated with insulin for 60 min at 100 nM. Insulin induced SPAK and NCC phosphorylation was impaired in WNK4 knockdown mpkDCT cells, although insulin increased phosphorylation of SPAK and NCC in negative control siRNA-transfected cells. B. Densitometry analysis of phosphorylation of SPAK and NCC by insulin in negative control and SPAK knockdown mpkDCT cells. Values (n = 4) are expressed as the ratio to the signals in insulin-free negative control siRNA-transfected cells.

Next, to confirm that SPAK is upstream of NCC in the phosphorylation cascade in this insulin-induced NCC phosphorylation mechanism, a SPAK knockdown experiment in mpkDCT cells was performed. As shown in Figure 3, insulin-induced NCC phosphorylation was impaired in SPAK knockdown mpkDCT cells, indicating that SPAK is upstream of this insulin-induced NCC phosphorylation. In addition, WNK4 knockdown was performed, to examine whether insulin-induced SPAK and NCC phosphorylation is diminished in WNK4 knockdown mpkDCT cells, since increased SPAK phosphorylation by insulin occurred at its specific phosphorylation site by WNK kinases. As expected, WNK4 knockdown in mpkDCT cells impaired the response of insulin-induced phosphorylation of SPAK and NCC, compared to negative control, clarifying that WNK4 is involved in this insulin-induced phosphorylation of SPAK and NCC (Figure 4).

**Figure 5:**
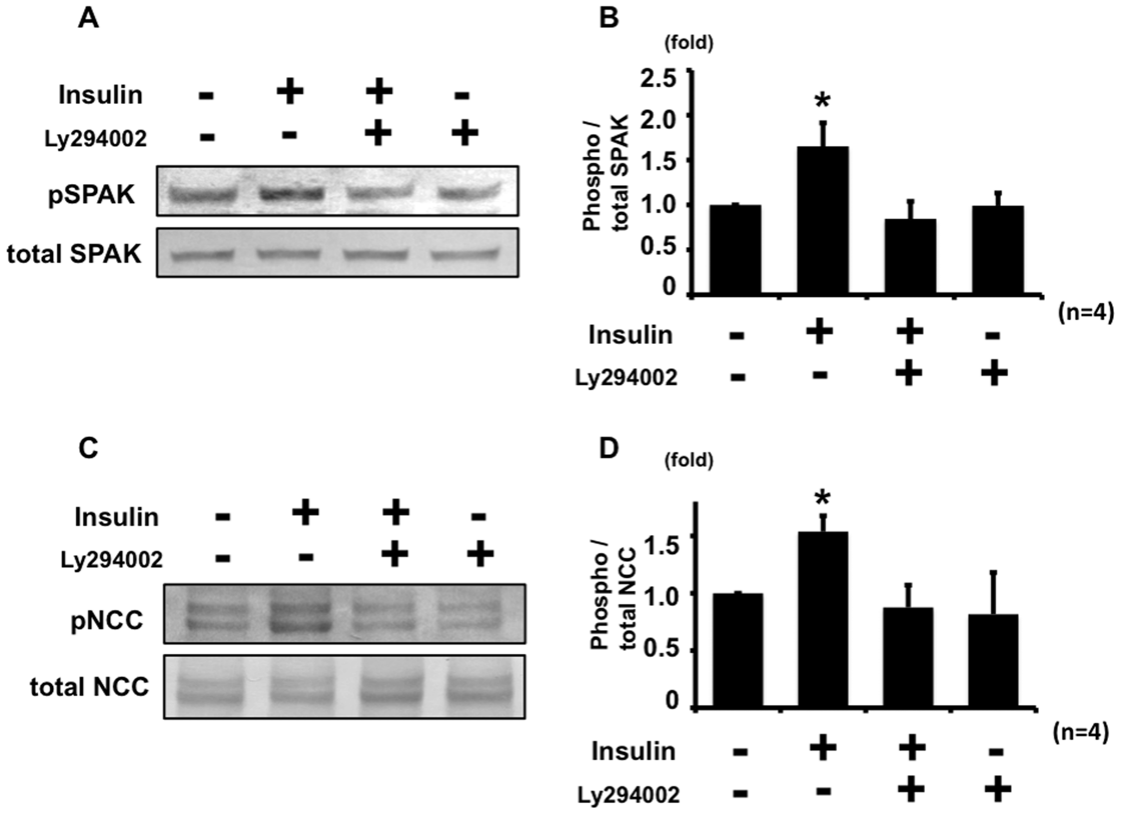
Insulin phosphorylates SPAK and NCC through PI3K in mpkDCT cells. Ly294002, a PI3K inhibitor, suppressed the insulin effect on SPAK (A, B) and NCC (C, D) phosphorylation in mpkDCT cells. Cells were incubated with insulin for 60 min and Ly294002 was added 30 min before insulin incubation. (A) and (C) show representative blots of phosphorylation of SPAK and NCC, respectively. (B) and (D) are densitometry analyses of phosphorylation of SPAK and NCC, respectively. The insulin effect on phosphorylation of SPAK and NCC was absent with Ly294002. Values (n = 4) are expressed as the ratio to the signals in insulin-free samples without Ly294002. *P<0.05.

As shown in Figure 1, insulin increased phosphorylation of Akt and SGK1 in mpkDCT cells, as well as SPAK and NCC. It is well known that insulin-induced phosphorylation of Akt and SGK1through phosphatidylinositol 3-kinase (PI3K) is required for their activation [20]. Since Akt and SGK1 have been reported to regulate sodium channel [8], [21], we hypothesized that the insulin effect on NCC phosphorylation could be through PI3K and Akt/SGK1 as well. Therefore, to determine whether PI3K is involved in this insulin-stimulated NCC phosphorylation, a PI3K inhibitor experiment was performed. Ly294002, a PI3K inhibitor, suppressed insulin-stimulated phosphorylation of SPAK and NCC (Figure 5).

These results indicate that insulin positively regulates phosphorylation of SPAK and NCC through PI3K in mpkDCT cell and that WNK4 is involved in this insulin-induced phosphorylation of SPAK and NCC.

### Insulin administration increased phosphorylation of OSR1, SPAK and NCC in the mouse kidney

**Figure 6:**
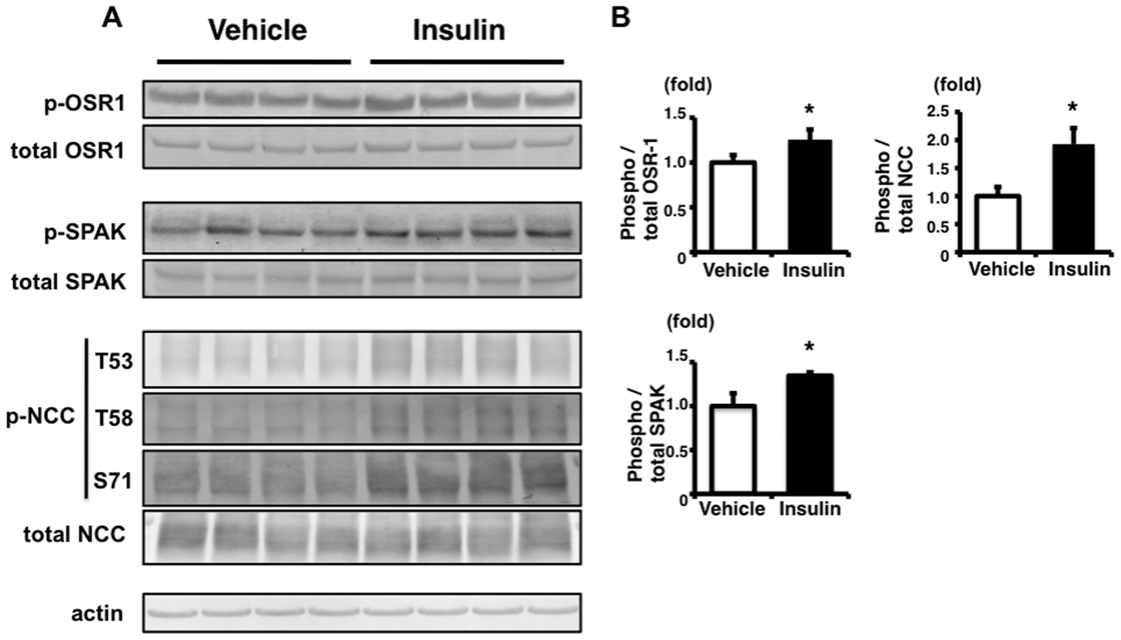
Insulin stimulation increases phosphorylation of OSR1, SPAK and NCC in mouse kidney. A: Immunoblots of phosphorylation of OSR1, SPAK and NCC in the kidney from insulin-stimulated mice on a normal diet. Phosphorylation of OSR1, SPAK and NCC was significantly increased at 90 min after insulin administration, compared to signals in the vehicle group. B: Densitometry analysis of phosphorylation of OSR1, SPAK and NCC in the kidney. In densitometry analysis, values (n = 4) are expressed as the ratio to the average of the signals in the vehicle group. *P<0.05.

**Figure 7:**
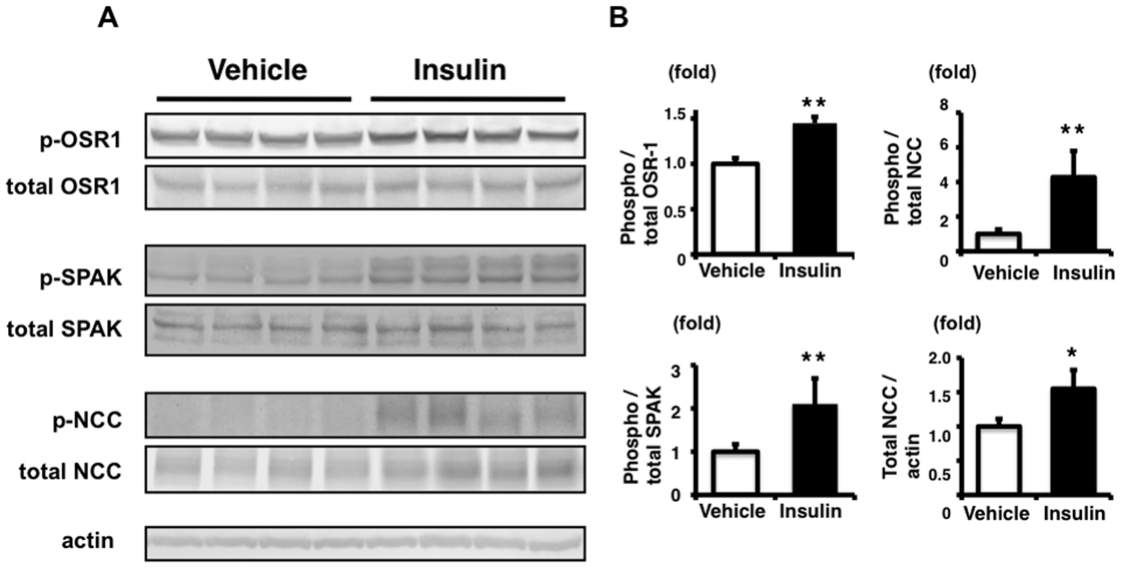
Increased phosphorylation of OSR1, SPAK and NCC in the kidney from mice on a high-salt diet. A: Immunoblots of phosphorylation of OSR1, SPAK and NCC in the kidney from insulin-stimulated mice on a high-salt diet. Phosphorylation of OSR1, SPAK and NCC was significantly increased at 90 min after insulin administration, compared to signals in the vehicle group. These differences between insulin-stimulated and control mice were more abundant and significant, compared with the normal diet experiment. As well as the phosphorylation of NCC, total NCC expression in the kidney was also increased in the insulin-stimulated group. B: Densitometry analyses of phosphorylation of OSR1, SPAK and NCC in the kidney. For densitometry analysis, values (n = 4) are expressed as the ratio to the average of signals in the vehicle group. *P<0.05, **P<0.01.

Next, mouse experiments were performed to investigate whether the NCC phosphorylation by insulin observed in the cultured cells was also functional *in vivo*. Insulin was intraperitoneally injected into C57BL/6 mice (5 U/kg), and OSR1, SPAK and NCC phosphorylation in the kidney was checked. As shown in Figure 6, insulin increased phosphorylation of OSR1, SPAK and NCC in the kidney, at 90 min after insulin injection. However, since the effect of insulin on OSR1, SPAK and NCC phosphorylation in the kidney from mice on a normal diet was slight, though it was significant, the same experiment was performed with mice on a high-salt diet. We expected that we could see the effect of insulin more easily, because phosphorylation of OSR1, SPAK and NCC is decreased in kidneys from mice on a high-salt diet, due to lower plasma aldosterone levels [4]. As expected, insulin more clearly increased phosphorylation of OSR1, SPAK and NCC in kidneys from mice on a high-salt diet (Figure 7). These results clearly showed that the insulin signal phosphorylates OSR1, SPAK, and NCC *in vivo* in mouse kidney, as well as in cultured cells.

**Figure 8:**
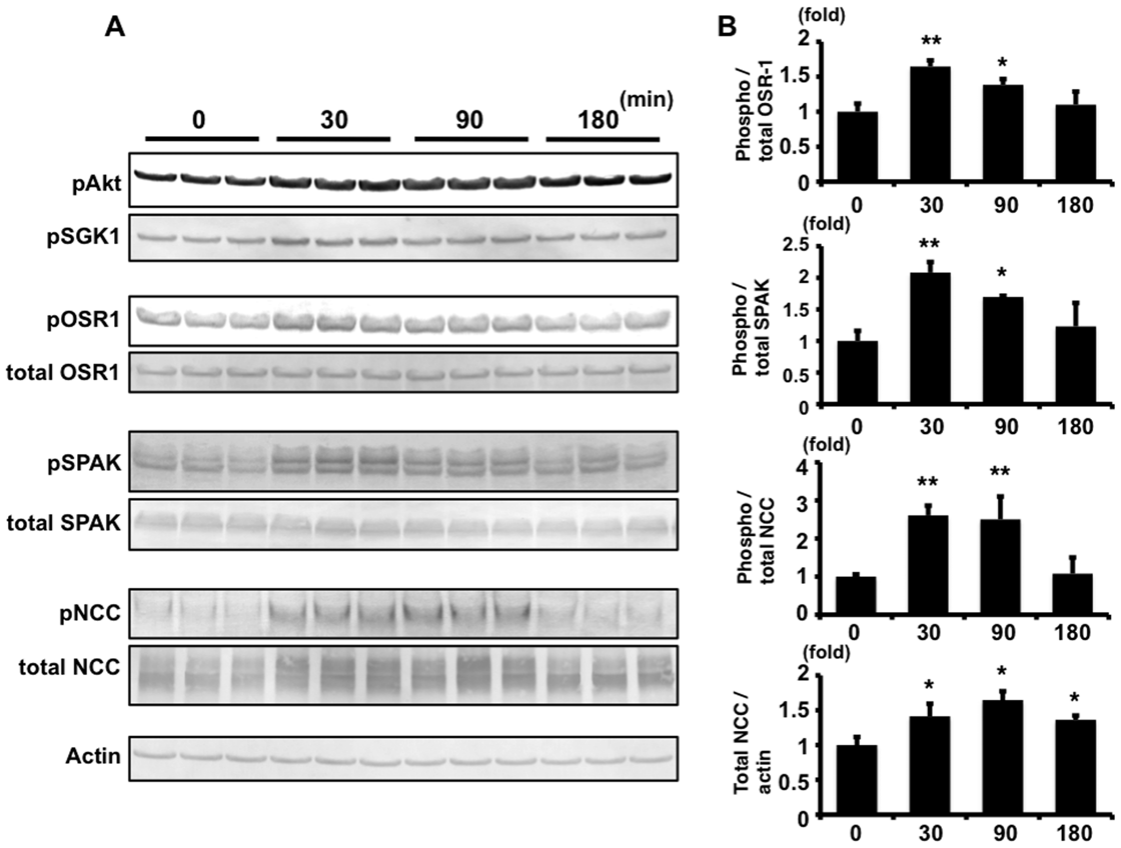
Time-course of insulin-stimulated OSR1, SPAK and NCC phosphorylation in insulin-stimulated mouse kidney. A. Immunoblots of the time-course of OSR1, SPAK and NCC phosphorylation in the kidney from insulin-stimulated mice on a high-salt diet. Thirty minutes after injection of insulin to mice, phosphorylation of OSR1 and SPAK increased to the maximal degree. NCC phosphorylation increased to 2.6-fold (p<0.01) at 30 min after insulin injection, and it was sustained for at least 90 min. Increased total NCC protein was also sustained in insulin-injected mouse kidney for least 180 min. Phosphorylation of Akt and SGK1 was also increased to the maximal degree at 30 min after insulin injection. B. Densitometry analyses of phosphorylation of OSR1, SPAK, NCC and total NCC protein in the kidney. For densitometry analysis, values (n = 3) are expressed as the ratio to the average of the signals in the vehicle group. *P<0.05, **P<0.01.

To evaluate the effect of insulin on the phosphorylation of OSR1, SPAK and NCC in the mouse kidney, a time-course experiment was performed (Figure 8). Thirty minutes after injection of insulin to mice, phosphorylation of OSR1 and SPAK increased to the maximal degree, and declined thereafter. Similar to the time-course of phosphorylation of OSR1 and SPAK, phosphorylation of Akt and SGK1 also increased to the maximal degree at 30 min after insulin injection.

The time-course of insulin-induced phosphorylation of NCC in mouse kidney was slightly different from that of OSR1 and SPAK. In mouse kidney, NCC phosphorylation increased to 2.6-fold at 30 min after insulin injection, and the level of NCC phosphorylation was sustained for at least 90 min, when phosphorylation of OSR1 and SPAK had already declined. Unlike SPAK and OSR1, total NCC protein was also increased in insulin-injected mouse kidney.

**Figure 9:**
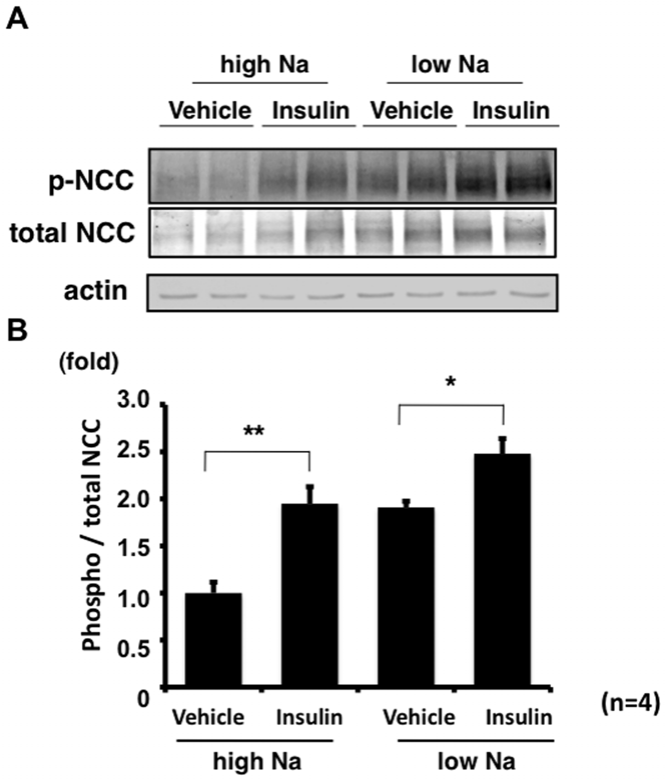
Effect of insulin on NCC phosphorylation in mouse kidney on high- and low-salt diets. A. Representative immunoblots of phosphorylated NCC in mouse kidney at 90 min after insulin administration to wild-type mice on high- and low-salt diets. B. In both the high- and low-salt diet groups, insulin administration significantly increased phosphorylated NCC. Moreover, acute insulin injection to the high-salt diet group resulted in a comparable level of NCC phosphorylation to the increased level of NCC phosphorylation in the kidney from the low-salt diet group due to a higher aldosterone level. For densitometry analysis, values (n = 4) are expressed as the ratio to the average of the signals in the vehicle control with the high-salt diet group. *P<0.05, **P<0.01.

To explore the physiological significance of insulin-induced NCC phosphorylation, NCC phosphorylation levels were compared in mice on high- and low-salt diets with or without insulin administration (Figure 9). Irrespective of salt intake, insulin-injected mice showed significantly increased NCC phosphorylation compared to control mice in both high- and low-salt diet-fed mice, suggesting that insulin action may be independent of aldosterone.

### SPAK is involved in the mechanism of NCC phosphorylation by insulin *in vivo*

**Figure 10:**
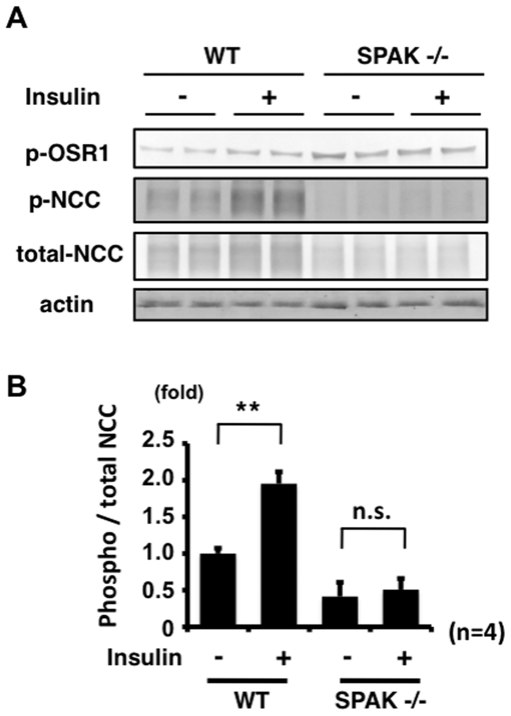
SPAK is involved in the mechanism of NCC phosphorylation by insulin. A. Representative immunoblots of phosphorylated NCC, at 90 min after insulin administration for to wild-type and SPAK knockout mice. An insulin effect on phosphorylation of NCC was not observed in SPAK knockout mouse kidney. B. Densitometry analyses of phosphorylation of NCC in the kidney from wild-type and SPAK knockout mice. For densitometry analysis, values (n = 4) are expressed as the ratio to the average of the signals in the wild-type vehicle group. **P<0.01. n.s. not significant.

It is well known that SPAK is a major kinase to phosphorylate NCC [17], [18], [22], [23]. In fact, as shown in Figure 3, insulin-induced NCC phosphorylation was impaired in SPAK knockdown mpkDCT cells. Therefore, to determine whether SPAK was involved in the mechanism of NCC phosphorylation by insulin *in vivo*, the same acute insulin injection experiment was performed on SPAK knockout mice [18]. Increased NCC phosphorylation by insulin was not observed in SPAK knockout mice compared to wild-type mice (Figure 10), indicating that SPAK is also involved in the insulin-induced NCC phosphorylation.

### WNK4 is involved in the mechanism of OSR1, SPAK and NCC phosphorylation by insulin *in vivo*

**Figure 11:**
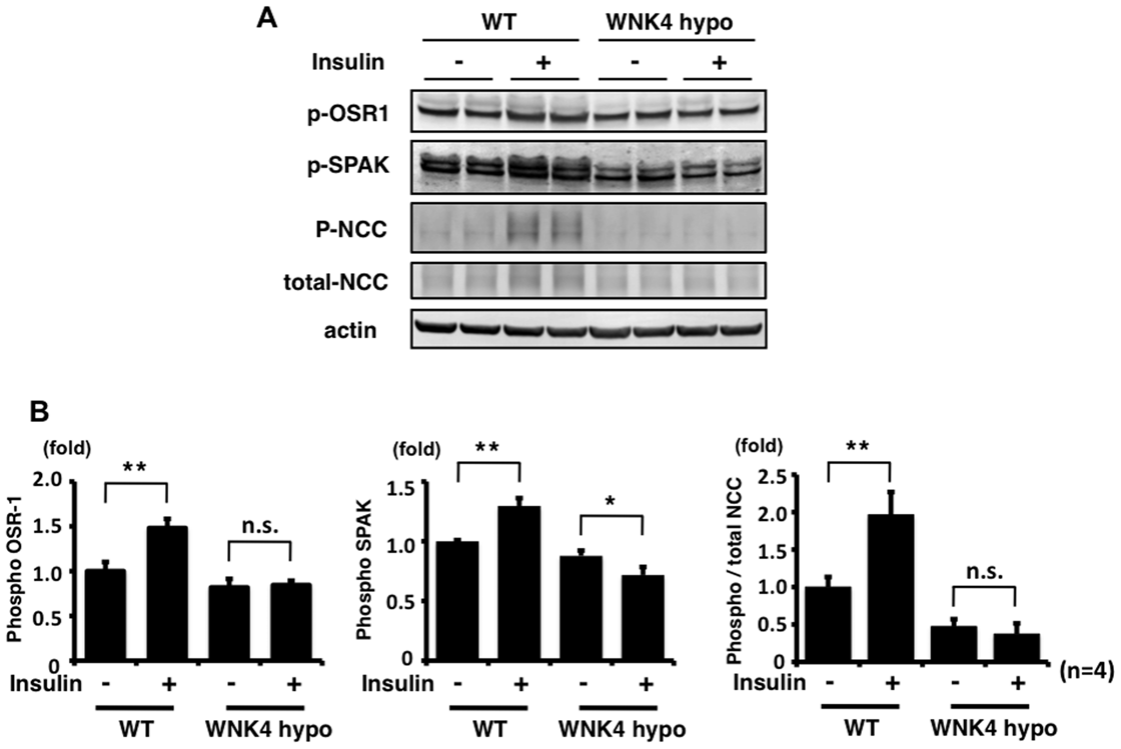
WNK4 is involved in the mechanism of NCC phosphorylation by insulin. A. Immunoblots of phosphorylated OSR1 and SPAK, at 90 min after insulin administration to wild-type and WNK4 hypomorphic mice. An insulin effect on phosphorylation of OSR1 was not observed in WNK4 hypomorphic mouse kidney. On the other hand, phosphorylation of SPAK was decreased significantly after insulin stimulation in WNK4 hypomorphic mouse kidney. B. Densitometry analyses of phosphorylation of OSR1, SPAK and NCC in the kidney from wild-type and WNK4 hypomorphic mice. For densitometry analysis, values (n = 4) are expressed as the ratio to the average of the signals in the wild-type vehicle group. *P<0.05, **P<0.01. n.s. not significant.

WNK4 knockdown in mpkDCT cells impaired insulin-induced phosphorylation of SPAK and NCC, as shown in Figure 4. Therefore, to confirm that WNK4 is involved in the insulin-induced phosphorylation of NCC *in vivo*, the insulin injection experiment was performed in WNK4 hypomorphic mice [24]. In WNK4 hypomorphic mice, increased OSR1, SPAK and NCC phosphorylation in the kidney was not observed with insulin stimulation (Figure 11), indicating that WNK4 is involved in the mechanism of the insulin-induced OSR1, SPAK and NCC phosphorylation *in vivo*.

## Discussion

Insulin is well known to regulate renal sodium reabsorption. It has been reported that insulin infusion increases sodium reabsorption in the kidney [9], [10], [11], [12]. However, a linkage between insulin and NCC has not yet been reported, although the effects of the renin-angiotensin-aldosterone system on NCC have been well documented [1], [4], [5], [6], [8], [25].

In this study, there was a linkage between insulin and NCC phosphorylation. To our knowledge, this is the first demonstration of insulin-induced NCC phosphorylation in a cultured cell line and *in vivo*. As shown in Figures 1 and 2, insulin increased the phosphorylation of SPAK and NCC in mpkDCT cells. OSR1, SPAK and NCC phosphorylation was also clearly increased in insulin-injected mouse kidney. In a time-course experiment, OSR1, SPAK and NCC were phosphorylated by insulin, but maximal NCC phosphorylation occurred later than maximal OSR1 and SPAK phosphorylation in mouse kidney (Figure 8). A similar phenomenon was also observed in the time-course experiment with mpkDCT cells (Figure 1). As previously clarified in SPAK kinase-dead knockin and knockout mice [17], [18], SPAK is upstream of NCC in the WNK-OSR1/SPAK-NCC phosphorylation cascade. Therefore, the present results demonstrated that SPAK phosphorylation occurs at first, and phosphorylated SPAK mediates NCC phosphorylation later, indicating that SPAK is upstream of NCC even in this insulin-stimulated NCC phosphorylation mechanism.

These findings suggest the involvement of SPAK in the mechanism of NCC phosphorylation by insulin. To confirm this, the same insulin experiment was performed in the SPAK knockdown cells and SPAK knockout mice (Figure 3 and 10). As expected, insulin-induced NCC phosphorylation was impaired in SPAK knockdown mpkDCT cells. Similarly, the increase in NCC phosphorylation by insulin was completely abolished in the SPAK knockout mice, supporting the essential role of SPAK in this insulin-induced NCC phosphorylation *in vitro* and *in vivo*. Involvement of WNK4 in insulin-induced NCC phosphorylation was also confirmed in this study. The insulin effect on phosphorylation of OSR1, SPAK and NCC was not observed in WNK4 knockdown cells and WNK4 hypomorphic mice. Taken together, these findings clearly show that WNK4 and SPAK are involved in the insulin-stimulated NCC phosphorylation mechanism *in vitro* and *in vivo*.

The detailed mechanism of WNK4 activation by insulin remains to be determined. It is clear that PI3K is involved in the insulin-induced NCC phosphorylation mechanism, as shown in Figure 5. PI3K is a key component of the insulin-signaling pathway. Insulin induces phosphorylation of Akt and SGK1 through PI3K, resulting in activation of Akt and SGK1 [20]. One possible mechanism is that insulin activates Akt/SGK1 and activated Akt/SGK1 modifies the function of WNK4, since WNK4 has several Akt/SGK1 phosphorylation motifs, and phosphorylation at one of these sites has been reported to alter WNK4 kinase activity [25], [26]. It is also possible that WNK1, as well as WNK4, might be involved in the NCC phosphorylation by insulin. WNK1 is reported as a substrate of Akt, a kinase strongly regulated by insulin, and Thr-60 of WNK1 is phosphorylated by insulin [27]. Activation of WNK1 could probably result in OSR1, SPAK and NCC phosphorylation by insulin as well.

To date, various regulators of NCC phosphorylation have been reported. Of these, there is no doubt that aldosterone is the major player for NCC phosphorylation *in vivo*. This aldosterone-dependent activation of NCC is physiologically important for the regulation of sodium excretion by sodium intake [4]. We also reported the angiotensinII effect on OSR1, SPAK and NCC phosphorylation, as well as that of aldosterone [7]. Thus, the effect of the renin-angiotensin-aldosterone system on NCC phosphorylation has been intensively investigated [1], [4], [5], [6], [8], [25]. On the other hand, the insulin effect on NCC has been poorly understood. It has been reported that insulin infusion increases sodium reabsorption by the kidney in both humans and animals [9], [10], [11], [12]. Although it has been reported that epithelial Na channel (ENaC) is regulated by insulin in mouse and rat kidneys [11], [12], the linkage between the other sodium transporters and insulin has not yet been investigated. Here, in this study, acute insulin infusion clearly increased NCC phosphorylation. We have reported that the WNK-OSR1/SPAK-NCC phosphorylation cascade is an aldosterone-dependent system [4]. A high-salt diet suppresses OSR1, SPAK and NCC phosphorylation due to lower plasma aldosterone levels. Inversely, a low-salt diet increases OSR1, SPAK and NCC phosphorylation. As shown in Figure 9, the acute insulin effect on NCC phosphorylation in the high-salt diet mouse kidney was equivalent to the low-salt diet effect on NCC phosphorylation, indicating that insulin may block the down-regulation of NCC phosphorylation by a high-salt diet. It is well established that the metabolic syndrome causes hyperinsulinemia [28], as a result of insulin resistance, and hyperinsulinemia causes an aberrant increase in sodium reabsorption by the kidney [9], [10], [28]. Interestingly, it has been reported that the metabolic syndrome enhances salt-sensitivity, which causes salt-sensitive hypertension [29], [30]. However, the mechanism responsible for this greater salt-sensitivity in hyperinsulinemic patients is still unknown. Our novel findings, that insulin diminished the down-regulation of NCC phosphorylation by a high-salt diet in the kidney, might be part of the explanation for increased salt-sensitivity in these hyperinsulinemic patients.

As shown in Figures 7 and 8, total NCC was increased in insulin-administered mice fed with a high-salt diet. Recently, we reported that total NCC was increased in *WNK4^D561A/+^* knock-in mice, a mouse model of PHAII [3]. On the other hand, total NCC was decreased in SPAK kinase-dead knock-in and knockout mice [17], [18]. Therefore, since we found that WNK4 and SPAK are involved in the insulin-induced NCC phosphorylation mechanism, increased total NCC in insulin-administered mice might be due to the same mechanism as in WNK4 and SPAK knock-in and -out mice. Unlike in mice fed with a high-salt diet, there was no significant increase in total NCC by insulin administration in mice fed with a normal diet (Figure 6). In the case of a normal diet, an increase in total NCC could be masked, because the basal expression level of total NCC is already increased due to lower aldosterone levels, as we previously reported [4]. In a previous study, chronic insulin-infused rats fed with a normal salt diet did not show increased total NCC, as seen in the present experiment involving mice fed with a normal diet, although thiazide sensitivity was increased in these rats, indicating that NCC was functionally activated [11], [12]. Examination of NCC phosphorylation in these rats may clarify the mechanism of increased thiazide sensitivity in insulin-infused rats, although further investigation is needed.

In summary, it was found that insulin increased phosphorylation of NCC in cultured cells and in mouse kidney *in vivo*. WNK4 and SPAK are involved in this insulin-stimulated NCC phosphorylation *in vivo*. The present findings might explain increased salt-sensitivity under hyperinsulinemic conditions.

## Materials and Methods

### Animal study

In acute insulin injection experiments, male mice were fed a normal NaCl diet, a high-NaCl diet (4.0% NaCl (w/w)) or a low-NaCl diet (0.01% NaCl (w/w)) for 7 days before insulin administration. All foods were obtained from Oriental Yeast Co., Ltd (Tokyo, Japan). Insulin was administered intraperitoneally at a dose of 5 U/kg, as previously reported [31]. Control mice received vehicle instead. Mice were sacrificed 90 min after insulin administration. In a time-course experiment, mice were fed a high-NaCl diet for 7 days and sacrificed 0, 30, 90 and 180 min after insulin injection. We used C57/BL6J mice for this acute insulin injection experiment. WNK4 hypomorphic mice [24] and SPAK knockout mice [18] were also used in this acute insulin injection experiment. These WNK4 hypomorphic and SPAK knockout mice were maintained on a C57/BL6J background. The protocols for this study were approved by the Institutional Animal Care and Use Committees of Tokyo Medical and Dental University (no. 10-176 and no. 100011).

### Immunoblotting

Semiquantitative immunoblotting was performed, as described previously [3], [32], to assess relative expression levels of proteins using whole kidney homogenate without the nuclear fraction (600 g) or the crude membrane fraction (17000 g). The intensity of bands was analyzed using Image J (NIH, Bethesda, MD). Rabbit anti-pNCC (Thr53) [4], rabbit anti-pNCC antibody (Thr58) [4], rabbit anti-pNCC (Ser71) antibody [3], rabbit anti-pOSR1 antibody [24], rabbit p-SPAK antibody [18], rabbit anti-OSR1 antibody [24], anti-SPAK antibody (Cell Signaling, Beverly, MA), anti-pAkt antibody (Cell Signaling), anti-pSGK1 antibody (Cell Signaling), and anti-actin antibody (Cell Signaling) were used as previously reported. In all the experiment except Figure 6, we used rabbit anti-pNCC (Ser71) antibody for detection of NCC phosphorylation. The specificity of total- and phospho-specific- SPAK antibodies was confirmed using SPAK knockout mouse (Figure S1). We also performed an absorption test of total- and phospho-specific- NCC antibody (Figure S2 and Figure S3), whose specificity was confirmed previously [3], [4].

### Cell culture

mpkDCT cells were cultured as previously reported [19]. Twenty-four hours before the experiments, the medium was changed to DMEM without FBS and insulin. mpkDCT cells were incubated with insulin (Sigma Aldrich) at 100 nM and lysed 60 min after insulin stimulation. In dose-dependent experiments, insulin was incubated at 20 nM and 100 nM. In a time-course experiment, cells were lysed 0, 5, 15, 60 and 300 min after insulin stimulation. Ly294002 (Sigma Aldrich) was added at 50 µM 30 min before insulin incubation. After incubation with reagent, the cells were lysed as previously reported [33]. In SPAK and WNK4 knockdown experiments, cocktails of 3 duplexes of siRNA for mouse SPAK and mouse WNK4 were obtained from the siTRIO library through COSMO BIO (Tokyo, Japan). The oligonucleotide sequences of the siRNAs were as follows: mouse WNK4 siTRIO SMF27A-2153-1 gca caa agc cca aca gcu uTT; mouse WNK4 siTRIO SMF27A-2153-2 gga aga uga ugg aga gaa gTT; mouse WNK4 siTRIO SMF27A-2153-3 cga cag agu ugu cga gug uTT; mouse SPAK siTRIO SMF27A-2154-1 ggu cag auc cau agggau uTT; mouse SPAK siTRIO SMF27A-2154-2 cgg aau aaa guc aga aaa aTT; mouse SPAK siTRIO SMF27A-2154-3 uga cau acg auu uga guu uTT. As a negative control, siTRIO negative control siRNA (COSMO BIO), which consists of cocktail of 3 duplexes of negative control sequence siRNA, was used. For knockdown, 50 nM of siRNA cocktail were used, and transfections were carried out using Lipofectamine RNAiMAX (Invitrogen). Experiments were performed at forty-eight hours after siRNA transfection.

### Statistical analysis

Statistical significance was evaluated using an unpaired *t*-test. P-values<0.05 were considered significant. When more than three groups were compared, one-way ANOVA was used, followed by Fisher's post hoc test.

## Supporting Information

Figure S1 **Conformation of total- and phospho-specific- SPAK antibody** ***in vivo*** **.** Immunoblot of kidney homogenate from wild-type (left lane) and SPAK knockout mouse (right lane) with our total SPAK and phospho-specific SPAK antibody. The disappearance of bands in the sample from a SPAK knockout mouse confirms the specificity of our antibody.
(TIF)

Figure S2 **Absorption test of total- and phospho-specific- NCC antibody for mpkDCT cell.** The specificity of these antibodies was confirmed previously [3], [4]. A. Confirmation of specificity of anti-pNCC (Ser71) antibody in a sample from mpkDCT cells. The signals detected by the antibody disappeared when the antibody was pre-incubated with antigen phosphopeptide (right panels), but they did not disappear with the corresponding non-phosphopeptide (middle panels). B. Confirmation of specificity of anti-NCC antibody in a sample from mpkDCT cells. The signals detected by the antibody disappeared when the antibody was pre-incubated with antigen.
(TIF)

Figure S3 **Absorption test of total- and phospho-specific- NCC antibody for mouse kidney.** A. Confirmation of specificity of anti-pNCC (Ser71) antibody in a sample from wild-type, WNK4 hypomorphic and SPAK knockout mouse kidney. The signals detected by the antibody disappeared when the antibody was pre-incubated with antigen phosphopeptide (right panels), but they did not disappear with the corresponding non-phosphopeptide (middle panels). B. Confirmation of specificity of anti-NCC antibody in a sample from wild-type, WNK4 hypomorphic and SPAK knockout mouse kidney. The signals detected by the antibody disappeared when the antibody was pre-incubated with antigen.
(TIF)
