# Supplementary figures and images for: Acute Insulin Stimulation Induces Phosphorylation of the Na-Cl Cotransporter in Cultured Distal mpkDCT Cells and Mouse Kidney
Source: PLoS One. 2011 Aug 31;6(8):e24277. doi: 10.1371/journal.pone.0024277 (PMC3164195; doi:10.1371/journal.pone.0024277)

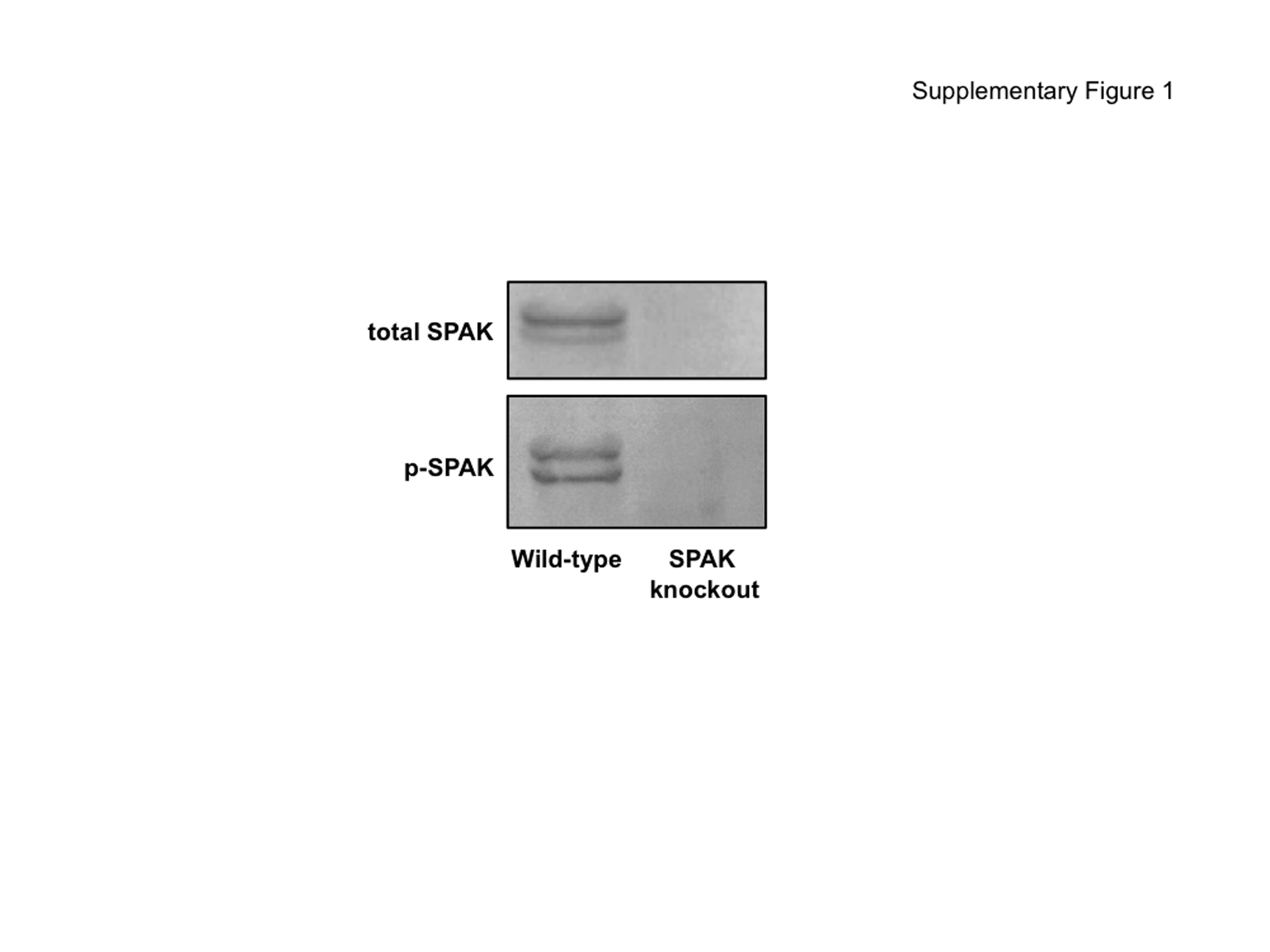

Supplement: Figure S1 — Conformation of total- and phospho-specific- SPAK antibody in vivo . Immunoblot of kidney homogenate from wild-type (left lane) and SPAK knockout mouse (right lane) with our total SPAK and phospho-specific SPAK antibody. The disappearance of bands in the sample from a SPAK knockout mouse confirms the specificity of our antibody. (TIF) [file pone.0024277.s001.tif]

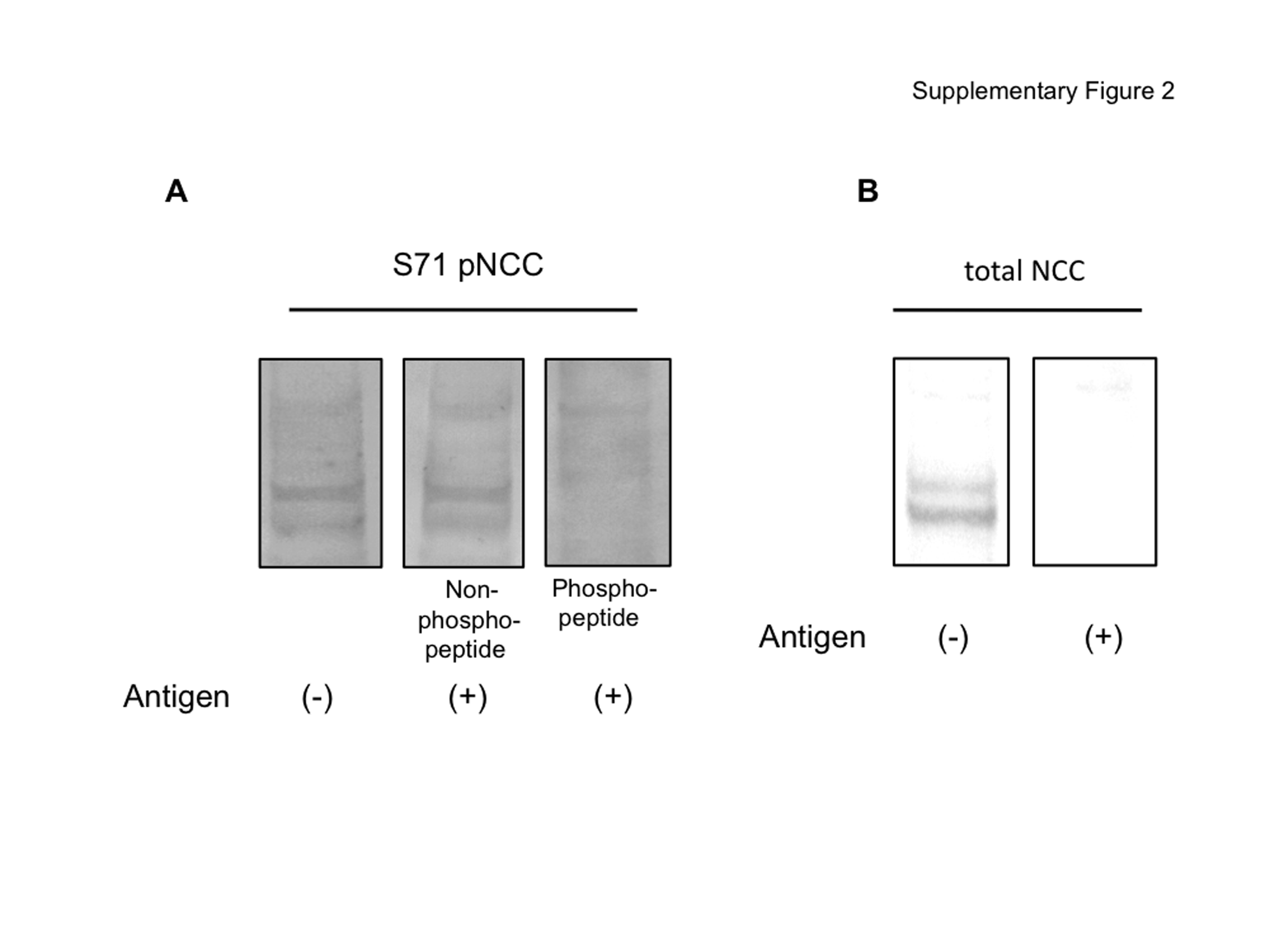

Supplement: Figure S2 — Absorption test of total- and phospho-specific- NCC antibody for mpkDCT cell. The specificity of these antibodies was confirmed previously [3], [4]. A. Confirmation of specificity of anti-pNCC (Ser71) antibody in a sample from mpkDCT cells. The signals detected by the antibody disappeared when the antibody was pre-incubated with antigen phosphopeptide (right panels), but they did not disappear with the corresponding non-phosphopeptide (middle panels). B. Confirmation of specificity of anti-NCC antibody in a sample from mpkDCT cells. The signals detected by the antibody disappeared when the antibody was pre-incubated with antigen. (TIF) [file pone.0024277.s002.tif]

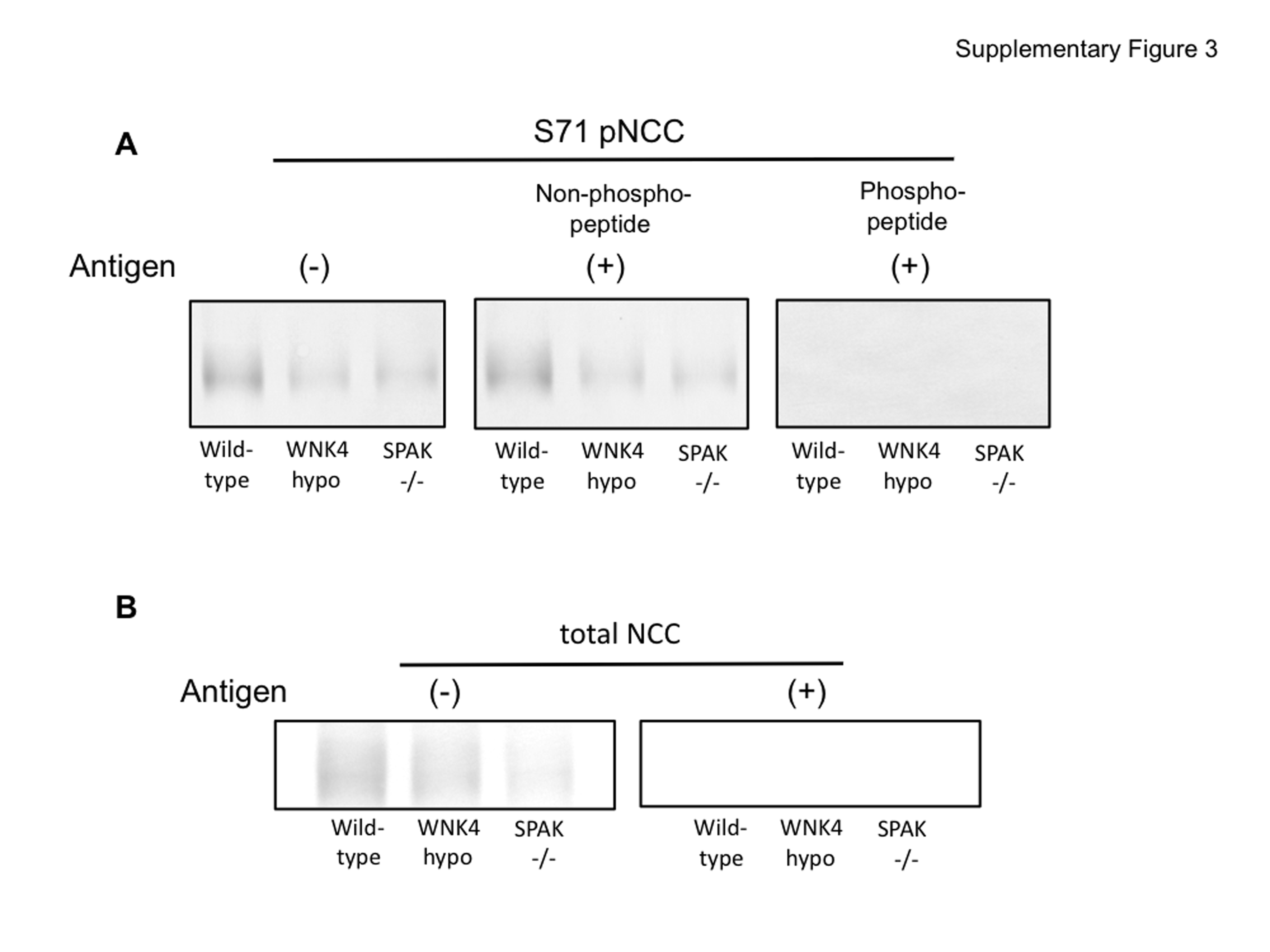

Supplement: Figure S3 — Absorption test of total- and phospho-specific- NCC antibody for mouse kidney. A. Confirmation of specificity of anti-pNCC (Ser71) antibody in a sample from wild-type, WNK4 hypomorphic and SPAK knockout mouse kidney. The signals detected by the antibody disappeared when the antibody was pre-incubated with antigen phosphopeptide (right panels), but they did not disappear with the corresponding non-phosphopeptide (middle panels). B. Confirmation of specificity of anti-NCC antibody in a sample from wild-type, WNK4 hypomorphic and SPAK knockout mouse kidney. The signals detected by the antibody disappeared when the antibody was pre-incubated with antigen. (TIF) [file pone.0024277.s003.tif]
